# Logical Development of the Cell Ontology

**DOI:** 10.1186/1471-2105-12-6

**Published:** 2011-01-05

**Authors:** Terrence F Meehan, Anna Maria Masci, Amina Abdulla, Lindsay G Cowell, Judith A Blake, Christopher J Mungall, Alexander D Diehl

**Affiliations:** 1 Mouse Genome Informatics, The Jackson Laboratory, Bar Harbor, ME, USA; 2 Dept. of Biostatistics and Bioinformatics, Duke University Medical Center, Durham, NC, USA; 3 Lawrence Berkeley National Laboratory, Berkeley, CA, USA; 4 Department of Neurology, University at Buffalo School of Medicine and Biomedical Sciences, Buffalo, NY, USA

## Abstract

**Background:**

The Cell Ontology (CL) is an ontology for the representation of *in vivo *cell types. As biological ontologies such as the CL grow in complexity, they become increasingly difficult to use and maintain. By making the information in the ontology computable, we can use automated reasoners to detect errors and assist with classification. Here we report on the generation of computable definitions for the hematopoietic cell types in the CL.

**Results:**

Computable definitions for over 340 CL classes have been created using a genus-differentia approach. These define cell types according to multiple axes of classification such as the protein complexes found on the surface of a cell type, the biological processes participated in by a cell type, or the phenotypic characteristics associated with a cell type. We employed automated reasoners to verify the ontology and to reveal mistakes in manual curation. The implementation of this process exposed areas in the ontology where new cell type classes were needed to accommodate species-specific expression of cellular markers. Our use of reasoners also inferred new relationships within the CL, and between the CL and the contributing ontologies. This restructured ontology can be used to identify immune cells by flow cytometry, supports sophisticated biological queries involving cells, and helps generate new hypotheses about cell function based on similarities to other cell types.

**Conclusion:**

Use of computable definitions enhances the development of the CL and supports the interoperability of OBO ontologies.

## Background

Biological databases use ontologies to connect common elements between disparate data sets to facilitate searching and analysis. These ontologies are structured hierarchies of logically connected classes intended to represent biological entities and their relationships to each other. These ontologies are often conceived of as graph-theoretic structures, with classes as nodes and relationships as edges. Biological ontologies must evolve to capture the ever-increasing complexity of data being generated by the life sciences community. The widely used Gene Ontology (GO), for instance, has grown from about 4,000 classes at its inception to its current form that encompasses nearly 30,000 classes and over 50,000 relationships [1]. Maintenance and development of such a large ontology is burdensome, a problem that is compounded by the creation of other ontologies with overlapping domains. In response to these concerns, the Open Biological and Biomedical Ontologies (OBO) Foundry was created to provide a set of principles to help standardize and coordinate a collection of ontologies to cover the biomedical domain [2]. One of these principles is that an OBO Foundry candidate ontology must have clearly specified and delineated content that is orthogonal to other OBO ontologies. Within the OBO Foundry, the Cell Ontology (CL) is the designated reference ontology for the representation of *in vivo *cell types from all biology [2].

First developed in 2004 [3], the CL is composed of about 1000 classes connected via *is_a *and *develops_from *relationships. The CL classifies cell types according to multiple criteria, including function, histology, lineage and taxonomy. These different modes of classification are vital for the users of the CL; however, the initial classification was done manually and not in accordance with commonly accepted ontology engineering principles such as normalization and modularity [4]. The resulting ontology contained numerous mistakes and omissions, and proved difficult to maintain and extend.

We have revised and extended the hematopoietic cell branch of the CL following a normalized approach in which computable definitions (sometimes called "logical definitions" or "cross-products") for classes are constructed in a modular fashion, using relationships to classes from other ontologies. These computable definitions can be expressed in ontology formats and languages such as OBO or OWL [2,5], and are treated as equivalence relationships between the defined class and some conjunction of classes. For example, the class "nucleate erythrocyte" can be defined as equivalent to the class of things that are both erythrocytes and that have a nucleus as a part. In OWL this can be written as:

"nucleate erythrocyte" EquivalentTo erythrocyte and has_part some nucleus where "erythrocyte" is declared in the cell ontology and nucleus is declared in the GO cell component ontology.

These computable definitions not only provide an integration point between ontologies, but they also allow tools called reasoners to automatically construct the ontology hierarchy (for example, between "nucleate erythrocyte" and "nucleate cell"). Our computable definitions all follow a genus-differentia structure, in which a class is defined by refining an existing more general class (the genus - in the above example this would be "erythrocyte") and one or more differentiating characteristics (the differentiae - in the above example this is the has_part relationship to nucleus) [6,7]. By adding disjointness constraints between classes in the ontology, reasoners can also detect errors in the graph structure. Two classes are disjoint if they cannot share any members. If during ontology construction a disjointness constraint is violated, the reasoner will flag the error. We also supply textual definitions that mirror the computable definitions, but are intended for human readers.

Other complex biomedical ontologies have employed computable definitions to support reasoning and help maintain the logical soundness of their structure including the Systematized Nomenclature of Medicine-Clinical Terms (SNOMED-CT), the Gene Ontology Next Generation Project (GONG), and the Ontology for Biomedical Investigations (OBI) [8-10]. The ontological structure of the GO has been improved in a similar manner by parsing class names into computable definitions; this approach originally added 223 missing relationships and is now routinely used in ongoing development [11]. Masci et al confirmed this process is well suited for cell types by using computable definitions in the design of a dendritic cell ontology consisting of 29 classes [12]. They defined dendritic cell types using cell surface expression of proteins as a major axis of differentia. Thus, a "CD8_alpha-negative, CD11b-negative dendritic cell" is a "conventional dendritic cell" (the genus) that "*lacks_plasma_membrane_part *CD8_alpha and CD11b" (the differentia). Masci et al found significant benefits in the amount and accuracy of information contained in the ontology compared to the information about dendritic cells available in the original implementation of the CL.

By integrating Masci et al.'s approach with our recent reorganization of the hematopoietic branch of the CL [13], we have created a new sub-ontology called "Hemo_CL". This ontology consists of over 330 hematopoietic cell classes (including 47 new additions) that have computable definitions generated using classes exclusively from ontologies in the OBO library. We demonstrate that computable definitions allow the use of automated reasoners to find mistakes in the ontology and to infer new relationships between classes. This approach also enriches other OBO ontologies by illuminating the need for new classes, and by highlighting ambiguous definitions of cell types in the scientific literature. Finally, an unexpected association between two classes highlights the potential use of computable definitions to generate new hypotheses regarding the nature of cell types.

## Results

### Linking to OBO library ontologies by computable definitions

**Figure 1:**
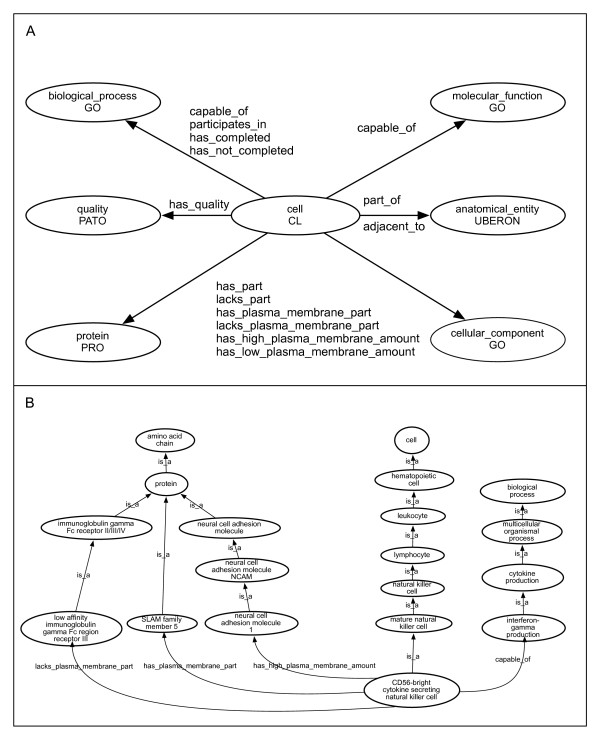
**Ontologies and relationships used in computable definitions**. (a) Ovals indicate the OBO Foundry ontologies whose classes are used in computable definitions with the CL. Arrows indicate the relationships used with a given ontology. The Protein Ontology and the cellular component of the Gene Ontology both use the 6 relationships describing expression. (b) Graphical representation of how cross product classes are used to define a natural killer cell type.

Our primary goal was to provide both computable definitions and human-oriented textual definitions for classes representing a wide range of hematopoietic cell types. In order to carry out this work independently of day-to-day editing of the Cell Ontology, we created a sub-ontology of hematopoietic cell types called Hemo_CL by taking the *is_a *descendants of the class "hematopoietic cell" and creating a new ontology. In this ontology, we manually specified computable definitions for 377 cell type classes (of 406 total classes). We assigned computable definitions using OBO-Edit [14], which allows the construction of simple genus-differentia definitions in the "cross-product" tab. By having computable definitions for the vast majority of classes, new relationships can be inferred between classes by the use of automated reasoners. The relations and OBO Foundry candidate ontologies used in these computable definitions are indicated in Figure 1a.

We make frequent use of the *has_plasma_membrane_part *relation as defined by Masci et al, in conjunction with classes from the Protein Ontology [15]. For example, a "CD4-positive, alpha-beta T cell (CL:0000624)" is defined as " a mature alpha-beta T cell that *has_plasma_membrane_part *CD4 (PRO:000001004). 278 cell type classes defined according to this template have been generated, reflecting the common use of flow cytometry to distinguish hematopoietic cells based on cell surface markers. In some cases, cell types are distinguished not by the expression of single protein on the surface but rather by a protein complex, in which case we use classes from the GO-Cellular Component ontology (GO-CC). For example, a "gamma-delta T cell (CL:0000798)" is defined as "a T cell that *has_plasma_membrane_part *gamma-delta T cell receptor complex (GO:0042106)". Certain cell types are defined by the absence of a cell surface protein, or by difference in the quantity of cell surface protein relative to other cell types [12]. In Figure 1b, the CD56-bright natural killer cell is partially defined as "mature natural killer" that lacks expression of one cell surface marker while expressing two other cell surface markers. One of these cell surface markers is expressed at higher levels than the geometric mean expression found for other leukocyte cell types and thus has a computable definition that uses the "*has_high_plasma_membrane_amount*" "*has_low_plasma_membrane_amount" *relationships as defined in Masci [12]. This example also illustrates how using computable definitions with curated ontologies eliminates the confusion that may result from the use of a synonym ("CD56" in place of "neural cell adhesion molecule 1") in the common name of a cell type class.

Some of our definitions also made use of negative criteria - for example, an alveolar macrophage is partially defined as a tissue-resident macrophage that lacks chemokine receptor CX3CR1 on its plasma membrane. In OWL this can be represented using a class-complement construct - however, there is no equivalent construct in the more limited obo format or the OBO-Edit tool. We circumvented this limitation by using OWL macros to expand *lacks_part *and *lacks_plasma_membrane_part *relationships to the semantically correct OWL expression (Table 1) [16]. In this way we can use OWL reasoners such as FaCT++ in combination with the OBO-Edit environment to detect a wider range of errors. For example, in an earlier version of the ontology several myelocyte classes were described as "*lacks_part *tertiary granule (GO:0070820)". Three levels up the *is_a *hierarchy, the ancestor class "granulocyte" had ten computable definitions including "*has_part *tertiary granule". Macro expansion found this and other violations of logic that are difficult to find manually because of the large number of computable definitions involved in ancestor terms.

**Table 1:** Translated OBO relationships used in Hemo-CL

OBO relationship	Simple OWL translation	Expanded OWL translation
*has_plasma_membrane_part*	has_plasma_membrane_part some ?Y	has_part some (GO:'plasma membrane' and has_part some ?Y)

*lacks_plasma_membrane_part*	lacks_plasma_membrane_part value ?Y	has_part exactly 0 (GO:'plasma membrane' and has_part some ?Y)

*capable_of*	capable_of some ?Y	bearer_of some (realized_by only ?Y)

*participates_in*	participates_in some ?Y	-

*has_completed*	has_completed some ?Y	transformation_of some (participates_in some ?Y)

*has_not_completed*	has_not_completed some ?Y	not transformation_of some (participates_in some ?Y)

Several of the OBO relationships used in Hemo-CL were translated into an OWL format. The left hand column contains the OBO-formatted relationships, the middle column contains relationships converted to a simple OWL format, and the right hand column contains expanded OWL-formatted relationships that employ negation and nested expressions. Details are in the methods.

Other OBO ontologies used in computable definitions are the GO-biological processes (GO-BP) [1], UBERON [17], and PATO [18]. The majority of computable definitions that contain GO-BP classes have the structure "D = *capable_of *a GO-BP". In the example presented in Figure 1b, the natural killer cell type is partially defined from its parent cell type by being capable of interferon gamma production. Here, the *capable _of *relationship states that instances of this cell type have the potential to produce interferon gamma given the right biological context, but it does not necessitate that all instances at all times are producing interferon gamma. In the cases of those few cell types that are characterized by having all instances at all times actively engaged in a biological process, the *participates_in *relationship is used. Pro-B cells, for example, are described as being in the "early stages of recombination of B cell receptor genes", so a computable definition using "*participates_in *immunoglobulin V(D)J recombination (GO:0033152)" is appropriate. Other cell types are characterized as having executed a process that changed their cell type. In these cases, the *has_completed *relationship is used as in a "mature alpha-beta T cell" is an "alpha-beta T cell that *has_completed *T cell selection (GO:0045058)". The *part_of *relationship is used with the species-neutral UBERON anatomy ontology when anatomical location is critical to defining a cell [17]. For example, a "Kupffer cell" is "a tissue-resident macrophage that is *part_of *hepatic sinusoid (UBERON:0001281)".

Clinical laboratories routinely identify granulocyte cell types both by flow cytometry and by staining characteristics. As we wanted to maximize the utility of the ontology, we decided to include both axes of differentia in our computable definitions. In the cases of granulocytes where cell types are differentiated based on nucleus or cytoplasm morphology, computable definitions are first generated between GO-CC and PATO [18] classes to create new cellular component classes. For example, "reniform nucleus" is defined as "nucleus (GO:0005634) that *has_quality *reniform (PATO:0001871)". Then basophilic metamyelocyte is defined as an "immature basophil that *has_part *reniform nucleus". The classes generated in this manner were given a separate namespace (cellular phenotype) and the prefix (CP) to distinguish them from the CL and are stored as part of the CL file. These classes may be incorporated into the GO or a phenotype ontology at a future date.

### Addition of species-specific cell types

**Figure 2:**
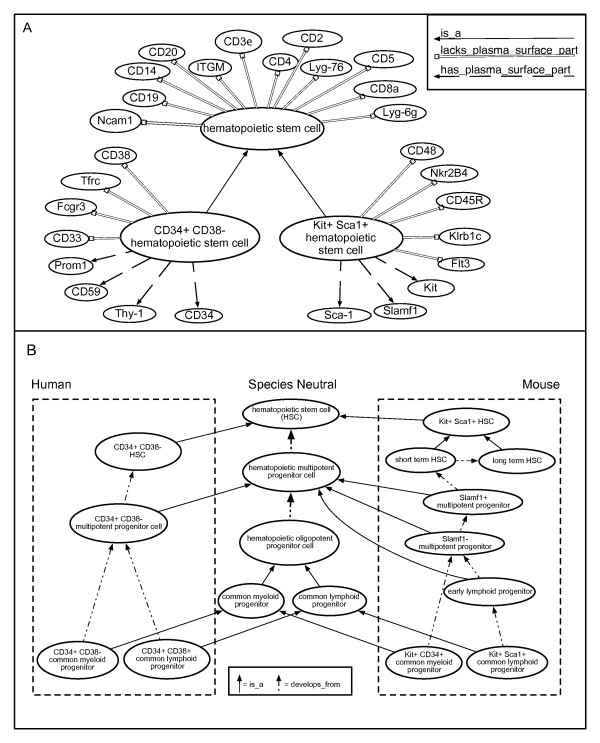
**Need for new cell types indicated during generation of computable definitions**. (a) The CL class "hematopoietic stem cell" and its computable definitions with the PRO ontology. New *is_a *subtypes of hematopoietic stem cell are created to represent species-specific expression of protein types. Abbreviations for protein types are used for clarity. (b) General hematopoietic progenitor cell types with characteristics shared by many species appear in the middle. Sub-types of progenitor cells classified by human-specific cell surface markers appear on the left and sub-types with mouse-specific cell surface markers appear on the right. Note that a developmental lineage of species-specific cell types can be determined by following the *develops_from *relationships.

Computable definitions for hematopoietic cell types highlighted the need for new classes to reflect species-specific differences. This was true for hematopoietic stem cell types (HSC) where there is little overlap of the cell surface markers between species. For example, the only similarity in cell surface markers between mouse and human HSC is that neither express cell surface proteins associated with fully differentiated immune cells (CD3epsilon, CD19, etc), a characteristic researchers call lineage-negative (lin^-^). Human HSC express CD34 and lack expression of CD38 on their cell surface whereas mouse HSC express other proteins, Kit and Spa-1, on their cell surface. To capture this in Hemo_CL, the species-neutral cell type "hematopoietic stem cell" is given computable definitions that define the lin^- ^characteristic. Depending on the research group and organism being studied, the number of lineage markers used differed from 3 to 19. In this case we chose a list of 12 markers that are used in both mouse and human stem cells to establish the lin^- ^phenotype (Figure 2a). From the species neutral HSC class, two *is_a *child classes are created for the HSC found in mouse and human. These classes are named for their distinctive cell markers as opposed to species names as it is probable that the human cell markers are the same for many primate species, as are mouse markers for rodents. Computable definitions to the specific markers are then made using the appropriate PRO classes and relationships. The transitive nature of the *is_a *relation means that properties used to define parent classes disseminate down through the child classes. As most of these computable definitions capture the expression of cell surface markers, this ontology should facilitate identification of these cell types by flow cytometry.

New cell types have been added to represent hematopoietic progenitor cell types that are currently an intense focus of research (Figure 2b). For Hemo_CL, progenitor cell classes are organized under multipotent progenitor, oligopotent progenitor, and lineage restricted progenitor cell types in a commonly used schema [19]. Multipotent progenitors develop from stem cells and have the ability to develop into any hematopoietic cell type but have limited self-renewal capability. Oligopotent progenitors develop from multipotent progenitors, are unable to self renew, and are classified by which hematopoietic cell types they can differentiate into. As with stem cells, there are species-specific differences in the surface markers that necessitate the use of more granular cell type classes. These species-specific cell types are placed under species neutral cell types but are connected to each other through *develops_from *relationships (Figure 2b).

### Ontology construction aided by computable definitions

**Figure 3:**
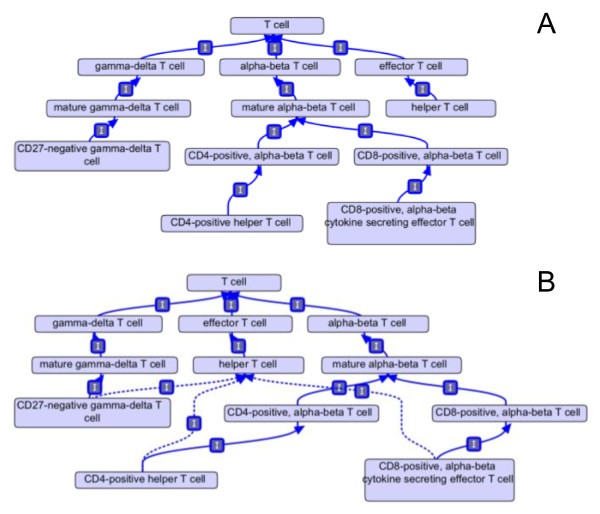
**Computable definitions allow automated reasoners to infer new relationships**. (a) Before reasoning, the class "helper T cell" on the right of the graph has no *is_a *sub-types. (b) Representation of the same entities after reasoning over the ontology. "Helper T cell" has numerous implied *is_a *sub-types based on its computable definition "helper T cell *is_a *T cell *capable_of *cytokine secretion".

Using the approach described here, classes are defined by refining a single parent class (the genus). We then use automated reasoners to build the full *is_a *polyhierarchy. This means that the asserted hierarchy follows the single inheritance principle, but the inferred hierarchy has multiple inheritance reflecting all the biological relationships between cell types. In Figure 3a, the cell class "helper T cell (CL:0000912)" has no *is_a *children in the asserted hierarchy. The computable definition for helper T cell is an "effector T cell that is *capable_of *cytokine production (GO:0001816)". When the ontology is reasoned over, four cell types are inferred to be *is_a *children to "helper T cell" because these cell types have computable definitions that meet the criteria of both effector T cell ("*has_completed *T cell selection, *capable_of *T cell chemotaxis") and helper T cell (*capable_of *cytokine production). The inferred links involving the gamma-delta T cell types were made because their computable definitions describe synthesis of specific cytokines such as "*capable_of *interleukin 17 production (GO:0032620)". Because these GO classes are *is_a *children to the GO class "cytokine production", the reasoner uses the logic of the GO to infer new relationships in the CL.

**Figure 4:**
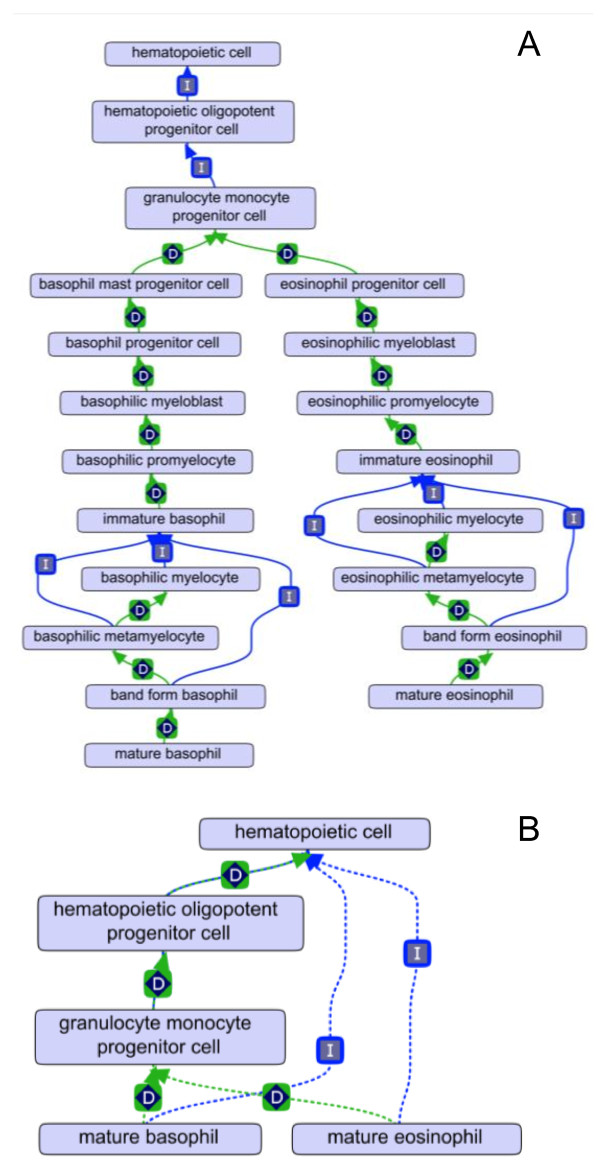
**Inferred relationships provide clarity within CL**. (a) A large number of classes have to be viewed to determine mature basophils and mature eosinophils develop from the same progenitor cell type in the asserted (i.e. unreasoned) hierarchy. D = *develops_from *relationship. (b) Inferred relationships by automated reasoning represent in a simple manner the shared progenitor cell type that mature basophils and mature eosinophils develop from. Dashed lines indicate inferred relationships.

The use of reasoners aided the analysis and editing of the Hemo_CL ontology. The transitive nature of the *develops_from *relationship allowed the reasoner to infer *develops_from *relationships between classes that are separated by multiple levels in the asserted *is_a *hierarchy (Figure 4). Mature basophils and eosinophils develop from a granulocyte monocyte progenitor cell type through seven distinct cell types that is convoluted to trace back in the asserted ontology (Figure 4a). An automated reasoner infers the *develops_from *relationship over the hidden cell-classes displaying the association in a more succinct and useful manner (Figure 4b). This ability is important in editing the ontology as it grows more complex. We also used automated reasoners to check for other errors in the ontology structure such as making two assertions between the same node pair using different relations. While not always logically inconsistent, this usually means some mistake has been made during construction of the ontology.

**Figure 5:**
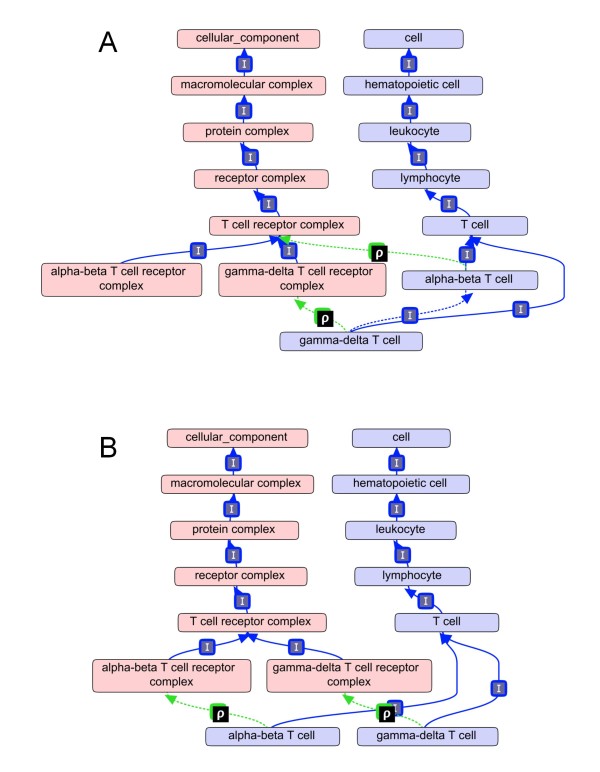
**Errors in manual curation are discovered with the use of automated reasoners and disjointness statements**. (a) Gamma-delta T cell type is inferred to by a sub-type of alpha-beta T cell, a violation of the disjointness relationship asserted between the two cell types (not shown). The inferred *is_a *relationship results from the too general cross product class "alpha-beta T cell *is_a *T cell that *has_plasma_membrane_part *T cell receptor complex". (b) Corrected version of the ontology where alpha-beta T cell is described as a "T cell that *has_plasma_membrane_part *alpha-beta T cell receptor". **ρ **= *has_plasma_membrane_part*

Errors that contradict biological knowledge were checked by using disjointness constraints. A reasoner will determine that a class is unsatisfiable if it is the *is_a *descendant of two disjoint classes. An example drawn from an earlier version of the ontology is shown in Figure 5a. The reasoner inferred that "gamma delta T cell" *is_a *type of "alpha beta T cell" based on their computable definition. However, these two classes are declared mutually disjoint and this combination of conditions gives rise to an inconsistency. This inconsistency resulted from the use of a too general differentia in the computable definition of alpha-beta T cell, "alpha beta T cell *is_a *T cell that *has_plasma_membrane_part *T lymphocyte receptor complex (GO:0042101)". Because the GO class "gamma-delta T cell receptor complex" *is_a *child to "T cell receptor complex" in the GO ontology, the reasoner inferred a gamma-delta T cell is a type of alpha beta T cell. Changing the differentia of the definition to "*has_plasma_membrane_part *alpha-beta T lymphocyte receptor complex (GO:0042105)" causes the reasoner to infer the two classes as separate and not violate the disjointness constraint (Figure 5b). In total, 28 *disjoint_from *constraints have been added to the ontology to ensure the computable cohesiveness of the structure. This approach also takes advantage of using the computable structure of a well-developed ontology (in this case, the GO) in the development of a less mature ontology.

### Commonality between classes discovered by automated reasoners

**Figure 6:**
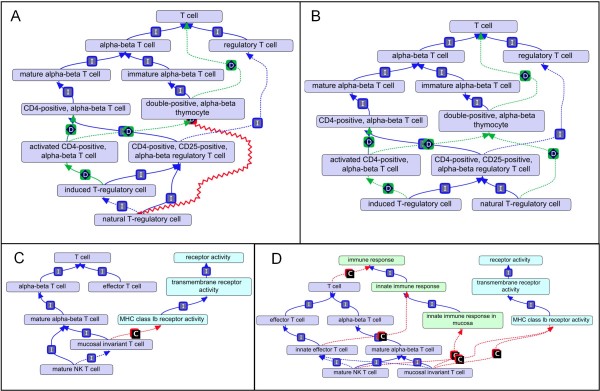
**Unexpected inferred relationships**. (a) Natural T-regulatory cell (nTreg) is inferred to be a type of induced regulatory T cell (iTreg). Red zig-zag line represents a redundant *develops_from *relationship as the reasoner infers both regulatory T cell (Treg) classes ultimately *develops_from *the same double-positive thymocyte class. (b) By using the *develops_from *relationship in the computable definition, Treg classes are defined by the type of class they directly *develop_from*. nTregs are no longer inferred to be an *is_a *sub-type of iTreg. (c) Mature NK T cell is inferred to be a sub-type of mucosal invariant T cell (MAIT). (d) Addition of an "innate effector T cell" class and refining of computable definitions for NK T cells and MAIT cells leads to new grouping of cell types by the reasoner. **C **= *capable_of*, Green = GO-biological process, Teal = GO-molecular function.

Computable definitions can reveal ambiguities in how a cell type is defined by a curator. One example is the cell types "natural T regulatory cell" (nTregs) and "induced regulatory cell" (iTregs). Because the cell types have the same *is_a *parent and share two cell surface markers, the reasoner inferred the former to be a subtype of the latter in an earlier version of the ontology, violating a disjointness constraint existing between the two classes (Figure 6a). Further investigation revealed that the only clear difference between the two cell types is that nTregs develop directly from double-positive thymocytes while iTregs develop from activated CD4 T cells [20]. Because activated CD4 T cells indirectly develop from double-positive thymocytes (DP thymocyte), the reasoner infers both regulatory T cell types are in the same developmental pathway and declares the *develops_from *relationship between the nTreg class and the DP thymocyte class to be redundant (as indicated by a red zig-zag in Figure 6a). To distinguish regulatory T cells based on the cell type they directly originate from, the *develops_from *relationship is removed from the asserted hierarchy and is instead used in the computable definitions. By declaring "induced regulatory T cell *is_a *regulatory T cell type that *develops_from *activated CD4 T cell", the reasoner logically infers that the two regulatory T cell types are separate sub-types (Figure 6b).

Some ambiguities resulted from how a cell type is defined in the scientific literature. The automated reasoning for the "mature NK T cell" provided an interesting example. Reasoning over the ontology failed to infer an *is_a *relationship between "NK T cell" and the "natural killer cell" despite "NK T cell" having the synonym "natural killer T cell" (not shown). Investigation of the literature revealed that the name "natural killer T cell" is controversial; the origin of the name "NK T" cell refers to cell surface expression of the NK1.1 protein and was not intended to imply a direct link to natural killer cells [21]. However some researchers feel there is enough functional overlap between "NK-" and "NK T-" cell types to justify the "natural killer T cell" moniker [21]. In Hemo_CL, the synonym is left in place as it reflects common usage, but NK T cell types and natural killer cell types are kept in separate branches of the ontology as inferred by the reasoner.

One relationship the reasoner does infer for mature "NK T cell" is that it is a sub-type of "mucosal associated invariant T cells" (MAIT) (Figure 6c). This result comes from both classes being alpha-beta T cell types that are *capable_of *interacting with MHC-Ib protein complexes through invariant T cell receptors. Investigating whether these two cell types should be grouped together, we hypothesized that other commonalities might exist between the two cell types. Upon review of the literature, we discovered some researchers have renamed the MAIT cells as "mucosal-NKT" cells based on the expression of NK1.1 by MAIT cells, in addition to their association with MHC-Ib family members [22,23]. Recently, two different research groups have demonstrated across species that MAIT cells are quickly activated and respond to bacterial antigens in a manner similar to NK T cells [24,25]. Following from these findings, we created an "innate effector T cell" class defined as "effector T cell *capable_of *innate immune response (GO:0045087)" (Figure 6d). The computable definitions for NK T cells and MAIT were refined to reflect their capability of participating in an innate immune response. When the ontology is reasoned over, NK T cells and MAIT T cells are grouped under this term (Figure 6d). This serves as an example of how a well-structured ontology that has computable definitions and is updated to represent the most current research can be used to generate hypotheses.

## Discussion

This work examines the advantages of using computable definitions to define cell type classes. We demonstrate that the use of automated reasoners coupled with computable definitions assist curators by automatically inferring the ontology hierarchy and detecting inconsistent statements. Computable definitions also highlight areas that do not represent the current state of knowledge, leading in this case to the addition of 47 new cell classes. Describing hematopoietic cell types with computable definitions using multiple ontologies strengthens the central role that CL has among the other OBO ontologies. Biological processes, protein expression, anatomical location, and phenotypic qualities are all integrated with the CL through computable definitions. This approach enriches other OBO ontologies by suggesting new classes for other ontologies when a sufficiently granular class needed for a computable definition is lacking. Creation of more granular CL classes also provides points of reference for generating more specificity in other OBO Foundry ontologies. As part of the generation of computable definitions, class requests were made of several of the OBO ontologies including the GO, PRO, and PATO. Such interactions increase the interoperability of the OBO Foundry ontologies.

Computable definitions for hematopoietic cell types have importance beyond ontology maintenance and integration for the wider scientific community. Sophisticated queries that incorporate cell types, protein expression, anatomical location, and biological processes are now possible. From Hemo_CL a user can inquire, for example, which hematopoietic cell types express a FoxP3 protein family member and are capable of regulating the immune system. Another type of query that Hemo_CL is particularly suited for is flow cytometry as cell markers is one of the major axes of differentia used to distinguish cells. We are currently setting up several collaborations to develop this use of the ontology further.

One potential benefit of logically defining cell types is the unexpected association of cell types generally not considered to be in the same grouping. We noted, in this regard, that mature NK T cells were inferred by the reasoner to be associated with mucosal invariant T cells, a view held by some [26] although not part of our initial representation. From this inference, we hypothesize that MAIT and NK T cells would have similarities in biological function. Similar hypotheses about the two cell types were developed by experts in the field, recently tested, and found to be true [24,25]. We believe hypothesis-generation represents a future use of well-structured biomedical ontologies that have computable definitions.

Our next focus for the CL is to examine and extend the representation of neuronal cell types including adding computable definitions. While the nervous system and the hematopoietic system are generally thought of as unrelated or even disjoint from each other (i.e. the immune privileged state of the nervous system) [27], there are intriguing commonalities among their cell types. The cell-to-cell contact that occurs between lymphocytes and antigen presenting cells has cellular structures similar to those in neuronal synapses (the "immune synapse") [28,29] and there is also expression of numerous immune proteins by neurons including MHC-I protein complexes that are important in neuronal plasticity [30]. By having computable definitions for these two branches, associations between cell types based upon biological function or protein expression may generate new hypotheses for testing neural-immune interactions.

## Conclusion

We generated new hematopoietic cell types to represent entities described in current research and added computable definitions for all hematopoietic cell types using classes from other OBO library ontologies. These computable definitions allow the use of automated reasoners to infer relationships between the classes based on the inherent logic. Unexpected inferred relationships are found upon investigation to result from mistaken or incomplete computable definitions of classes, and in at least one case reflect the ambiguous use of the term in the biomedical literature. The inferred relationships also permit placement of cell classes into functional groups without the problems associated with multiple inheritance in an asserted hierarchy. Requesting new classes needed for a computable definition also enriched other ontologies. Finally, we believe inferred relationships between branches of the CL will lead to unexpected associations between disparate areas of biology and help in the generation of new hypotheses.

## Methods

A sub-ontology of the CL called Hemo_CL was created by taking the *is_a *descendants of hematopoietic cell type and creating a new ontology. This new ontology is available at:

http://obo.cvs.sourceforge.net/viewvc/obo/obo/ontology/anatomy/cell_type

(note that this ontology has been integrated into the main CL ontology, but a record of the Hemo_CL subset as described in this paper will remain in the source control system. The revision number described in this paper is hemo_CL.obo revision 1.47).

Computable definition generation used a method modified from that described by Masci et al, where 29 dendritic cell types were redefined as cross-product classes [12]. Computable definitions take the form "a C *is_a *G that D" where C is the hematopoietic cell class being defined, G is the more general class and D is the set of differentia that characterize instances of C from instances of D. The general class is the asserted *is_a *parent to the class being defined. Differentia (D) are defined as relationships to other class classes that reside in the OBO Foundry ontologies Gene Ontology (GO), Protein Ontology (PRO), Uber Anatomy Ontology (UBERON), Phenotypic Quality ontology (PATO), and CL. As cell types are considered independent continuants [2], only relationships that include this entity in their domain are used. Relationships used include those from the Relationship Ontology (RO) and its proposed extension, and the *capable_of *relationship described in our previous publication [13]. The computable definitions for the 29 dendritic cell types described by Masci et al [12] containing the relationships *has_disposition *and *has_function *were converted to a definition containing the relationship *capable_of*.

For reasoning we use a combination of OBO-Edit and the OBO-Edit reasoner with Protégé 4 and OWL reasoners. We use a standard translation to OWL in which OBO format relationships are translated to SomeValuesFrom expressions in OWL by using the NCBO OBOinOWL mapping option that is part of OBO-Edit. In order to take advantage of OWL constructs such as negation and nested class expressions from within OBO format and OBO-Edit we use a macro expansion strategy. First we translate to OWL, then we selectively expand certain expressions as depicted in Table 1. The expansion of the *capable_of *relation does not affect reasoning but is required for compatibility with BFO. All the relations used in the expansions are defined in the new version of the RO ontology. Directly expressing the semantics of *has_low_plasma_membrane_part *and *has_high_plasma_membrane_part *is outside the scope of OWL. We adopt the formal textual definitions described in Masci et al [12] for *has_low_plasma_membrane_amount *and *has_high_plasma_membrane_amount*, but cannot provide an expansion rule that directly expresses the semantics in OWL.

Ontology editing was performed using OBO-Edit 2.1. Both the rule-based and the link piler reasoners were used to reason over the ontology. A MIREOT strategy [31] was used to bring in selected subsets of external ontologies into the hemo-CL ontology (this is implemented in OBO-Edit 2.1 using the *save is_a closure *option in the save menu). Figures are either screenshots from OBO-Edit 2.1 (Figures 3, 4, 5 and 6) or are Graphviz images exported from OBO-Edit and modified by Omnigraph Professional 5.2.1 (Figures 1 and 2).

## Abbreviations

CL: OBO Cell Ontology; GO: Gene Ontology; GO-BP: GO biological process; GO-CC: GO cellular component ontology; PRO: Protein Ontology; OBO: Open Biological and Biomedical Ontology; HSC: hematopoietic stem cells, OWL: Web Ontology Language; PATO:Phenotype and Trait ontology

## Authors' contributions

TFM did the majority of the ontology development and wrote the manuscript, AMM contributed terms for granulocytes and dendritic cells and assisted in revision of the text, AA optimized the OBO-Edit software tools for the needs of the work, LGC and JAB provided critical input and assisted in revision of the manuscript, CJM assisted with the construction of computable definitions and revision of the text, and ADD directed the work and assisted in revision of the text. All authors have read and approved the final manuscript.
